# Supplementary material for: Are carbon emissions trading and green financial instruments synergistic? -Comprehensive quantitative research based on content analysis
Source: PLoS One. 2024 Mar 7;19(3):e0298601. doi: 10.1371/journal.pone.0298601 (PMC10919601; doi:10.1371/journal.pone.0298601)
Supplement: S1 Data — (ZIP) [file pone.0298601.s001.zip › Data/地级市Z_F工作报告.zip!/╔╜╬≈/┬└┴║╩╨/└┤╫╘╣1⁄2╓┌║┼í╛┬φ┐╦╩2╛▌═°í┐_2006.docx]

各位代表：

现在，我代表市人民政府向大会作工作报告，请各位代表连同《吕梁市国民经济和社会发展第十一个五年规划纲要（草案）》一并审议，并请各位政协委员和其他列席人员提出意见。

一、“十五”时期及2005年经济和社会发展回顾

“十五”时期，我们在省委、省政府和市委的正确领导下，坚持以科学发展观统领全局，紧紧围绕“三新”奋斗目标，全面实施“一主三化”发展战略，大力推进改革开放，不断引深结构调整，着力建设和谐吕梁，经济和社会各项事业取得了可喜成就。

——综合实力显著增强。全市地区生产总值达到30095亿元，年均增长133%，人均地区生产总值首次突破1000美元。财政总收入达到6008亿元，年均增长4297%，由全省第8位攀升为第6位。

——发展质量不断提升。农业综合生产能力继续提高，粮食单产比“九五”时期提高149%；产值在5000万元以上的农副产品加工龙头企业发展到5户。煤、焦、钢铁等三大支柱产业规模快速扩张，链条双向延伸，占工业总产值的比重达到80％。新型建材、电力、化工、服务、劳务等产业的发展步伐明显加快。三次产业的比例由184:467:349调整为67:60:333。

——发展环境逐步优化。法制建设加强，依法行政进程加快，服务效率提高，对外开放成效显著。交通、水利、电力等基础设施建设取得重大突破，全市铺开并建成300余处重点市政工程，建成区面积扩展了一倍多。吕梁作为区域中心城市的框架初步形成。

——社会事业长足进步。人才强市战略深入实施，科技进步对经济增长的贡献率达到35%；基础教育水平不断提高，“普九”任务全面完成，职业教育和成人教育有了新的成效，吕梁学院筹建工作全面启动；群众性体育活动广泛开展，基层文化建设和文化艺术创作取得新成果；公共卫生体系建设全面推进，食品药品放心工程全面开展，抗击“非典”和防控禽流感工作取得了重大胜利；高度重视安全生产和环境保护，安全生产被动局面得到有效扭转。市区空气质量二级以上天数达到225天。

——人民生活水平逐步提高。就业社保和救灾救济工作顺利推进，城乡困难群众的基本生活得到初步保障。全市城镇居民人均可支配收入达到8130元，年均增长1655%；农民人均纯收入达到1991元，年均增长1162%。“十五”时期成为建区以来群众得实惠最多的时期之一。

各位代表，通过五年的努力，我们已全面完成了“十五”计划预期的各项目标任务。这些成绩的取得，凝聚了吕梁全体干部群众的智慧与心血。在此，我代表市人民政府，向各位人大代表、政协委员、各民主党派、工商联和社会各界人士以及全市人民，向驻吕省直单位、解放军指战员、武警官兵和政法干警，向所有关心吕梁、支持吕梁发展的同志们、朋友们表示衷心的感谢和崇高的敬意！

承前启后的2005年，我们坚持以科学发展观统领全局，主要做了以下工作：

（一）以经济结构调整为主线，着力推进新型能源化工和原材料工业基地建设

项目建设方面，继续坚持大上项目、上大项目、上好项目，实施重大调产项目173个，完成投资104亿元。其中115个工业调产项目已有81个建成投产，15个部分投产，完成投资8371亿元，新增产值55亿元。全市工业增加值达到1117亿元，同比增长26%，工业税收对财政收入贡献率达到714%。

园区建设方面，在交城夏家营、文水百金堡、汾阳三泉等“省示范工业区”的带动下，孝义梧桐、中阳尚家峪工业园区迅速提升，方山大武、岚县普明、交口双池、离石信义、兴县西川和临县三交等工业项目区建设全面起步。纳入统计范围的七个工业园区全年完成工业总产值1374亿元、工业增加值474亿元，分别占到全市的42%、43%，引领产业集群发展的态势日益凸显。

培育龙头企业方面，继续坚持大项目牵动战略，先后引进香港建滔集团、大连机床集团、山东兖矿、法国欧罗福集团等新的战略投资者，离柳焦煤集团、中钢集团、大土河焦煤集团等本地企业集团也都有新的骨干项目开工。40户重点监控企业产值占到全市工业总产值的639%，其中7户企业跨入全省百强行列。

发展循环经济方面，通过政策引导、市场驱动、典型带动，鼓励企业链条化组合、清洁型生产、循环式发展，一个以煤焦化、煤电铁钢、煤电建材等产业链条为重点的“资源互用、产业联结、首尾相顾、变废为宝、利益互动”的循环经济发展模式正在形成。

（二）以增加农民收入为核心，着力夯实扶贫攻坚和新农村建设的基础

农业产业化进程加快。小杂粮、马铃薯基地面积占到总播种面积的5246％；肉牛育肥基地、绒肉羊基地规模分别达到20万头、120万只；新增以红枣、核桃为主的经济林面积20万亩；反季节蔬菜达到45万亩。一批农副产品龙头企业发展壮大。

劳务输出和扶贫移民形成亮点。全年培训21个专业的技工148万人，转移129万人；转移农村剩余劳动力近50万人，人均劳务收入458元。建成25个移民新村，12万农民搬出了山庄窝铺。

农村基础设施建设步伐加快。全年完成317个村村通水泥路(油路)4841公里，村通电话率达到95%，广播电视覆盖率达到94%。

（三）以中心城市建设和管理为龙头，着力推进城乡统筹一体化进程

中心城市建设力度不断加大。吕梁市区龙凤南大街南延、滨河南路东延、滨河北路拓宽改造等一批城市交通网络工程全面竣工，“三山”绿化、集中供热二期、污水处理厂、垃圾处理厂等一批公益性工程建设进展顺利。各县市区积极开展城市管理年活动，狠抓市政建设，取得了明显成效。

服务业发展步伐持续加快。交通运输、房地产、信息咨询、商贸物流、餐饮娱乐、文化休闲等城市产业迅速发展，全市倾力打造自然风光游、酒都文化游、黄河古镇游、革命传统游等旅游产品，全年旅游综合收入达到824亿元，同比增长2591%，旅游业正在成为吕梁的新兴产业和城市名片。

小城镇和小康示范村建设富有特色。各县市区把小城镇建设与工业园区建设、旅游开发、民营企业发展和中小学布局调整有机结合、统筹推进，一批有较强辐射带动能力的工业强镇、文化名镇、商贸集镇、旅游大镇迅速崛起。汾阳贾家庄、孝义西王屯、中阳太高等一批小康示范村成了全市新农村建设的样板。

（四）以路水电等基础设施建设为突破口，着力缓解加快发展的瓶颈制约

去年10月28日太军高速公路汾离段胜利通车，同日离柳段奠基开工；忻黑线、大交线和岢大线部分路段改扩建工程全面完成；沿黄公路完成79公里路基工程；307线、209线、沿黄干线公路、石清线等国省道建设和改造工程已列入投资计划。横泉水库成功截流，引文入川工程近期即可开工。柳林电厂二期工程全面开工，三期工程前期工作已经展开；建成煤气、矸石综合利用电厂11个，新增装机容量129万千瓦；临县220千伏输变电工程全面完工，文水220千伏输变电工程顺利开工。

（五）以软环境建设为抓手，着力实现招商引资和对外开放新突破

深入推进行政审批制度改革，对全市行政许可项目进一步清理，对行政许可主体资格、许可事项、法律依据、办理程序等进一步规范。特别是政风行风评议和市级行政审批集中办理取得重大成效，企业和群众办事难的问题得到缓解。

继续坚持“大招商、招大商、招好商”的思路，不断扩大对外经贸合作和交流。先后组团对陕西、内蒙、河北、上海、天津、辽宁等省市区进行了经贸考察和招商引资；参加了4次全国性招商引资洽谈会，成功举办了中国吕梁第三届红枣节；与山西焦煤集团签订了300亿元的一揽子投资协议；成功举办了2005中国吕梁政银企座谈会，签订3713亿元的贷款协议；组建了城市建设投资公司，搭建了政府与开发银行战略合作的平台。

（六）以构建和谐吕梁为目标，着力推进民主法制建设和各项社会事业发展

继续健全完善村民自治和企业民主管理制度，积极推行政务、厂务和村务公开。继续加强社会治安综合治理，严厉打击各类违法犯罪活动。不断加大科技开发力度，引进吸收推广科技示范项目500项，科技进步对经济增长的贡献率比上年提高了5个百分点。积极实施农村中小学布局调整和寄宿制学校建设，继续推进中小学人事制度改革。大力开展教师培训，完成中小学校危房改造164万平方米。兴县、石楼、临县通过了“普九”达标验收，全市“普九”任务全面完成。188万名贫困学生享受到了“两免一补”政策的实惠，高考达线首次突破4000人大关。新建疾病防控中心和传染病院27个，三级卫生保健网络正在形成。成功举办了吕梁市第一届运动会和多项国家级赛事，展示了体育事业蓬勃发展的新形象。社会保障工作有序推进，养老保险、医疗保险和失业保险参保人数分别比上年增加527％、424％和136％；救灾救济工作稳步扎实，困难群众和弱势群体的生活问题得到了有效保障。农村计划生育四项奖励政策全面落实到位，长效节育措施深入落实，人口自然增长率控制为583‰。

（七）以推进依法行政为切入点，着力加强政府自身建设

全面贯彻落实《行政许可法》和《全面推进依法行政实施纲要》，进一步规范和完善行政执法责任制，积极推进行政效能建设，全面加强执法人员法律和业务培训，严格推行持证上岗、亮牌执法制度，政府社会管理和公共服务法制化水平进一步提高。在强化资源保护、环境治理、安全生产监管等管理领域作了新的探索，矿产资源有偿使用制度、环境污染限期治理和挂牌督办制度、安全生产一票否决等制度得到认真落实，“一企一矿绿化一山一沟”治理模式广泛推广，政府统筹经济社会、人与自然和谐发展的能力得到提高。

市政府主动向市人大及其常委会报告工作，自觉接受人大监督，积极支持人民政协工作，加强同民主党派、无党派人士和群众团体的联系，广泛听取各方面的意见和建议。全年共办理人大、政协转来的议案、提案521件，反馈率达100%，办理满意率在80%以上。

认真开展保持共产党员先进性教育，全面加强政府系统廉政建设和反腐败工作。严格执行“会审、联签、民评”和“公款报销公开、职务消费公示、公务接待限额”等六项制度，继续纠正医药购销、医疗服务、教育收费、公路“三乱”等行业的不正之风，纠正处理了162名国家机关工作人员和国有企业负责人的煤矿入股问题，依法清理了16亿元的拖欠工程款，维护了政府依法行政、廉洁从政的形象。

在肯定成绩的同时，我们也清醒地看到，经济和社会发展中还面临着许多困难和问题。主要表现在以下几个方面：贫困人口和低收入人口仍占全省的一半以上，扶贫攻坚任务还相当艰巨；结构性矛盾突出，产业层次较低，资源浪费和环境污染问题比较严重，支柱产业抗御市场风险能力不强；社会事业发展滞后，特别是农村教育、科技、文化、卫生等事业的发展水平与社会主义新农村建设的要求有很大差距；基础设施欠帐很多，建设步伐依然滞后，不能满足经济社会发展的需要；城镇化水平不高，城市承载和带动能力弱，“脏乱差”问题还没有得到解决；安全生产形势严峻，平安稳定工作需要进一步加强；部分政府公务人员宗旨意识不牢，工作作风不实，服务水平和行政效率亟待提高。这些问题的存在，使我们忧心如焚、寝食难安。我们惟有采取得力措施加以解决，才能孚民望、得民心，才能有资格在这块古老而凝重的土地上，肩负起时代赋予的重任!

二、“十一五”时期的经济社会发展指导思想、目标体系和战略重点

今后五年是我市加快发展、科学发展、和谐发展的关键时期，面对的将是机遇与挑战并存，总体有利发展的大环境。从国际看，经济全球化深入发展，科技进步日新月异，生产要素流动加快，尤其是以跨国公司为主体的产业重组在全球范围内展开，这为我市承接国际国内的产业和资本梯次转移创造了条件。从国内看，我国经济经过宏观调控步入稳定增长期，新一轮经济增长对能源原材料的需求将稳定增长，国家全面实施“中部崛起”战略，我市11个县区有望列入“西部开发”范围，将赢得更多政策支持。从我市看，丰富的地下资源继续成为吸纳资本的优势，基础设施改善拉近了对外经济技术合作的距离。持续数年的经济结构调整，使我市经济总量迅速扩张，发展水平显著提高，为加快发展奠定了较为坚实的基础。因此，我们必须切实增强机遇意识，进一步解放思想，创新思路，励精图治，乘势而上，努力开创我市“十一五”经济和社会发展的新局面。

根据市委一届三次全会精神，“十一五”时期，我市国民经济和社会发展的指导思想是：以邓小平理论和“三个代表”重要思想为指导，以科学发展观统领全局，坚持“一主三化”发展战略，努力实施大项目、大开放、大环境“三大工程”，突出抓好以增加农民收入为重点的扶贫开发和新农村建设、以循环经济为重点的经济结构调整、以中心城市为重点的特色城镇化建设、以路水电为重点的基础设施建设、以招商引资为重点的改革开放、以教育科技文化卫生为重点的社会事业发展等重点工作，着力打造“九大产业链”，切实完善人才、资金、软环境、社会化服务四大保障体系，实现稳定脱贫，建设新型能源化工和原材料工业基地，构建经济繁荣、人民富裕、社会和谐、环境友好的新吕梁。

按照上述指导思想，“十一五”期间我市将采用新的指标体系指导、推动全市经济社会发展。规划指标分为预期性和约束性两大类。预期性指标，是预计和期望达到的目标，主要通过引导市场主体来实现；约束性指标是必须实现的目标，主要通过依法加强管理和提供服务来实现。这一指标体系，由经济增长、社会发展、科技进步、资源环境、人民生活等五个方面的46项指标组成。这里，我着重就三个方面的8项指标作一说明。

一是关于经济增长速度问题。《纲要（草案）》提出，今后五年全市地区生产总值年均增长15%以上，人均地区生产总值年均增长14%以上，两项指标均高于全省预期目标。这是综合考虑各方面因素，着眼于加快发展的需要和可能提出的。“十五”时期，全市地区生产总值年均增长133%，高出了我们的预期，而且在经济结构调整的持续推进下，我市已经建成和铺开了一大批重大项目，奠定了较好的基础。我们认为这个目标是积极的，经过努力是可以实现的。

二是关于节能和环保问题。《纲要（草案）》提出，今后五年全市万元地区生产总值综合能耗下降20%以上，年均下降44%；万元地区生产总值主要污染物排放量下降35%，年均下降83%。这是针对资源环境压力日益加大的矛盾提出的，具有明确的政策导向，体现了建设资源节约型、环境友好型社会的要求。这两项指标均比全省预期目标低5个百分点，这是综合考虑我市属于重型结构地区，产业层次比较低，节能和环保压力很大的实际确定的。尽管要实现这一目标难度很大，但是我们有决心、有信心完成。

三是关于教育、卫生和科技问题。《纲要（草案）》提出，今后五年全市高中阶段毛入学率达到80%，县乡村三级医疗机构达标率达到80%，新型农村合作医疗覆盖率达到90%以上，高新技术产业增加值占生产总值的比重达到8%。这些目标的提出，目的在于引导全市各级把工作重心放在解决涉及群众切身利益的问题上，更加注重经济社会协调发展，更加注重城乡统筹发展，更加注重依靠科技进步，让广大人民群众充分享受我市科学发展、和谐发展的成果。

各位代表，“十一五”期间我们将改变主要根据经济总量论政绩的考核办法，从今年开始把五个方面46项指标全部纳入各级各部门的考核体系，严格考核。我们将咬定目标，殚精竭虑，攻坚克难，奋力开拓，全面完成既定的目标，让宏伟的蓝图变成壮丽的现实，让一个充满生机和活力的新吕梁崛起在三晋大地!

今后五年全市要紧紧围绕以下战略重点做好工作。

一是加快扶贫开发和新农村建设。扶贫开发是吕梁各项工作的重中之重，新农村建设是“三农”工作的总抓手。我们要站在以民为本和全面建设小康社会的政治高度，完善“以工补农、以财扶农、以城带乡”的开发式扶贫思路，采取更加有效的攻坚措施，决战扶贫攻坚。要扭住农民增收和改善农村环境两个重点，大力发展现代农业，加强农村基础设施建设，加快农村社会事业发展，深化农村综合改革，推进农村民主政治建设，努力提高农村经济社会发展水平。

二是引深经济结构调整。按照“开放引进、开发拉长、多元做大、科学发展”的思路，发挥资源优势，依靠科技进步，广泛利用资本市场，着力打造煤焦化、煤电铁钢、煤电铝、煤电建材、酿造、农副产品加工、旅游、商贸服务、新兴产业和高新技术等九大产业链条，促进资源综合利用、产业升级上档，着力把我市经济推上内涵式发展、集约型增长的轨道。

三是推进特色城镇化建设。坚持走以中心城市带动的特色城镇化路子，经过五年努力把全市城镇化率提高到35%以上。市区建设要着眼长远、科学规划、突出特色、提升品位，以开放的思维、市场化运作的方式，培育一批支撑城市发展的产业，建设一批完善城市功能的工程，努力把吕梁建成承东启西、独具特色的区域性中心城市，建成黄河东岸的一颗璀灿明珠。各县市也要科学规划、合理布局，继续加快城镇化进程，提高城市建设与管理水平，努力做到处处有亮点，年年有变化，整体提高吕梁城镇化建设水平。

四是突出基础设施建设。要敏锐把握国家加大中西部地区基础设施建设力度的机遇，进一步创新投融资方式，创优建设环境，狠抓重点工程，统筹功能配套，尽快建成一批同经济社会发展水平相适应的交通、水利、电力等骨干工程，从根本上消除基础设施的瓶颈制约。

五是深化改革扩大开放。以攻坚的精神深化改革，以创新的思维扩大开放，从经济政治文化社会各个领域入手，以改革促开放，以开放促发展，着力解决影响发展和稳定的深层次问题。改革要抓住症结，加快步伐，超越部门利益，科学谋划，依法推进；开放必须全方位、高水平，彻底敞开吕梁门户，科学划定准入门槛，通过对外开放引进资金、引进项目、引进技术、引进人才、引进管理、引进理念、引进创新模式等我们缺少的各种要素。

六是大力发展社会事业。在推动经济发展的同时，创新发展思路，调整支出结构，把更多的精力、更大的财力投入到社会事业的发展上，特别要高度重视科技进步和农村教育、卫生事业的发展，切实加强安全生产、环境保护、公共卫生等领域的管理，认真解决城乡弱势群体的实际困难，让更多的人享受到改革发展的成果和公共财政的“雨露”。

各位代表，“十一五”期间的战略目标，承载着全市353万人民的希冀与重托，完成这些目标将是一次历史性的壮举。人民鞭策着我们，事业呼唤着我们。我相信，有党的政策的指引，有全市干部群众的智慧和力量，我们的目标一定要实现，我们的目标也一定能够实现!

三、2006年的主要工作任务

各位代表，今年是“十一五”规划的开局之年。做好今年的工作，对于全面实现今后五年的奋斗目标，加快建设新吕梁的进程具有十分重要的意义。

根据“十一五”规划的总体安排，我们确定2006年经济和社会发展的主要预期目标是：

地区生产总值增长15%；

单位地区生产总值综合能耗下降56%；

单位地区生产总值主要污染物排放总量下降83%；

财政总收入增长20%；

工业增加值增长165%；

全社会固定资产投资总额增长25%；

社会消费品零售总额增长13%；

居民消费价格总水平上涨控制在3%以内；

外贸进出口总额增长12%；

农民人均纯收入增长125%；

城镇居民人均可支配收入增长12%；

城镇新增就业岗位223万个，城镇登记失业率控制在3%以内；

吕梁市区二级以上天气总数达到233天；

人口自然增长率控制在7‰以内。

实现上述发展目标，我们要重点做好以下十个方面的工作：

（一）大力抓好“项目推进年”，夯实加快发展的产业基础

抓好现有项目确保经济增长。牢固树立“项目吕梁”的理念，把总投资436亿元的100个工业项目作为重中之重，力争年内完成投资100亿元以上。重点支持山西晋兴能源2000万吨矿井、中钢80万吨高速线材、文通钾盐二期等42个项目，续建交城金桃园240万吨矿井改造、中钢240万吨炼钢、孝义城财180万吨轧钢等23个项目，新开工柳林联山40万吨二甲醚和200万平方米微晶玻璃等35个项目，确保全年工业总产值达到390亿元，增长18%。

抓新上项目培育竞争新优势。把握宏观政策走向和市场形势的变化，引导现有企业按照循环经济的理念，科学统筹上项目。依托资源优势，引入战略投资者，开发和引进一批延伸产业链条、填补产业空白的龙头骨干项目，年内力争使太钢、山西焦煤集团“一揽子”项目和孝义、离石的甲醇项目等后续项目取得实质性进展。按照分类管理、责任到部门的原则，分解落实各类项目推进的目标任务，千方百计让项目规划、投资协议落到实处。对落实下来、迟迟未开工的项目，逐一摸底，分析症结，拿出对策，尽快启动，特别是对提升产业档次、增加公共产品供给的项目要优先扶持，大力推进，以培育持续发展、协调发展的新力量。

抓项目管理凝聚发展合力。建立以县为主、区域推进的项目管理制度，坚持属地管理的原则，把中央、省属项目和地方项目统一纳入推进计划，一体化服务。成立分管副职任组长的行业项目组，建立四大班子领导直接联系项目，包干负责的工作制度。建立项目推进联席会议、季度交账和督查检查制度，确保项目推进工作取得实效。

（二）推动产业结构上档升级，转变经济增长方式

以引进促整合，推动资源规模化开发节约化利用。坚持发挥资源优势和节约利用资源并重，引导资源、技术、资金等生产要素向大企业、大集团集中，形成规模化开发、节约化、深度化利用的竞争优势，实现资源价值的最大化。煤炭行业，按照“关小、改中、上大”的思路，加快资源整合步伐，通过大企业、大集团对小矿进行联营改造、兼并参股，总体上提高煤炭工业集中度、竞争力和安全生产水平。焦炭行业，根据资源禀赋和环境承载能力，合理调控生产规模，引导企业调整组织结构，改变生产模式，组建生产联合体，向链条式生产和联合销售转变，扭转受资源和市场两头挤压的被动局面。冶金行业，扶持现有轧钢项目，调整产品结构，鼓励建设1000m3以上炼铁高炉，坚决淘汰不符合产业政策和环保要求的小高炉；依托铝土资源优势，对已经立项和正在争取的孝义宏基100万吨、交口肥美80万吨等氧化铝项目，择优扶持，加快我市氧化铝工业发展步伐。制造行业，重点扶持孝义大连机床集团铸造、交城上海德力西集团电器等项目；抓住晋沪两地区域性合作，共同建设山西上海工业园区的机遇，积极争取在我市建设上海大众汽车集团的配件分厂。

以开发促延伸，争创支柱产业发展强势。紧紧围绕九大产业链条，深入推动产业结构优化升级。尤其要充分发挥我市优质主焦煤的资源优势，以建设国家级煤化工生产基地为目标，大力发展煤化工业。围绕“肥、醇、炔、苯、油”五条主线，科学整合焦炭副产品，引导化工项目合理布局，重点在焦油加工、粗苯精制、煤液化上求突破。全力抓好宏特公司100万吨焦油深加工、山西晋兴能源100万吨煤变油两个项目的前期工作，推动文通盐桥5万吨硝酸钾、6万吨氯化铵、375万吨硝酸铵，交城宏特5万吨延迟焦、14万吨炭黑等10个重点项目尽快开工建设。

以发展循环经济促环保治理，再现吕梁蓝天碧水。全面启动《吕梁市环境综合整治三年规划》，制定实施建设节约型社会的实施办法和考核评价体系。引导煤炭、焦化、冶炼、电力等行业改进生产工艺和技术装备，循环利用废弃物和排放物，推行清洁生产；实施新上项目环保审查和“三同时”制度；加强水资源管理，推进节水型社会建设，加强对重点河流的监控，全面启动通道绿化、交通干线两侧荒山绿化、村庄绿化、厂矿区绿化、环城绿化、城市绿化六大造林绿化工程，努力使全市环境状况明显好转。

以园区化促产业集群，打造引领产业发展“主力舰队”。已初步建成的工业园区要着力抓提升，正在起步的要高起点、高标准推进，努力把这些园区建设成为传统产业的提升基地、新型产业的示范基地、高新技术的孵化基地、民营企业的创业基地。以园区集聚大企业，重点培育“三个方阵”的32户大企业、大集团，通过培育大企业提高产业集中度，使这部分企业成为引领全市经济发展的“主力舰队”。

以新型产业促多元化发展，培育后续产业。加快建设柳电二期2×60万千瓦坑口电厂和联盛、中钢、大土河等一批煤矸石、煤气发电项目，壮大电力工业。支持利虎玻璃、康欣生物制药等新兴产业项目，使其尽快成为新的经济增长点。支持汾酒集团深化产权制度改革，重振酒都雄风。支持红枣、核桃、沙棘、小杂粮等龙头企业创品牌、拓市场，真正把产品优势转变为经济优势。利用太中(银)铁路、太军高速公路建成后形成的区位优势，及早谋划建设区域性物流中心，积极发展商贸流通、文化休闲、餐饮旅游、信息中介等行业，全面提升服务业的发展水平。

（三）创新“三农”工作思路，加快扶贫开发和社会主义新农村建设

提高特色农业产业化水平。在切实加强耕地保护的同时，充分利用我市气候和生态资源，全面实施雁门关生态畜牧区、沿黄红枣带、吕梁山东西两麓核桃杂粮带、平川果菜畜牧和农产品加工带的“一区三带”建设规划，加快特色农产品区域性基地建设。突出优质核桃林栽植和红枣烘干房建设两个重点，通过财政补助引导群众发展核桃林，力争年内新增10万亩；集中安排扶贫资金和财政支农资金，在沿黄枣区再建1300间红枣烘干房，形成日烘干261万公斤的能力，解决红枣雨季霉烂问题。抓住全省倾斜扶持太行山、晋西北革命老区的有利时机，做好项目筛选上报工作，尽最大努力争取省政府扶持。

完善扶贫开发方式。以南北五县为重点，继续狠抓产业扶贫、整村推进、移民搬迁和劳动力培训转移为主的开发式扶贫，力争解决10万贫困人口的温饱问题。按照“下山、出沟、进镇、沿路”的思路，坚持整村搬迁、就近安置、自愿迁移的原则，完成省下达的12200人的扶贫移民任务；继续抓好农村劳动力转移培训工作，争取新增劳务输出6万人。

稳步推进社会主义新农村建设。根据中央关于建设社会主义新农村“20字方针”，科学制定新农村建设规划，按照“两培育、五创建”的总体要求，以“治五乱、达五化、建三室”为切入点，组织实施“十村示范、百村试点、千村治理”工程，培养典型、示范引路。以产业开发、村镇建设为重点，稳步推进农村住房、用水、用电、沼气、道路、通讯、广播电视等基础设施建设；把新农村建设与工业园区建设结合起来，大力推广中阳钢厂等企业“一矿带一村、一企带一村”的新农村建设模式，把企业壮大同农村产业培育、基础设施建设、公益事业发展结合起来，引导更多的企业投入到社会主义新农村建设中来。

创新支持“三农”工作的长效机制。全面落实党在农村的各项方针政策，坚持“多予、少取、放活”的方针，重点在“多予”上下功夫。坚持“以财扶农”，本着存量适度调整，增量重点倾斜的原则，按照中央“三个高于”的要求，加大财政投入的力度。不折不扣地落实“三减免、三补贴”的惠农政策。创新金融服务方式，拓展农业信用担保、农业保险等业务，增加对“三农”的信贷投放。狠抓“以工补农”，落实省政府提出的“以煤扶农”的政策措施。

（四）加快中心城市建设，推进特色城镇化进程

集中力量抓好市区建设。以建设省级卫生文明城市为目标，以城市净化、绿化、美化为主题，借助于城投公司这一平台，多渠道筹集建设资金，全面实施龙山、虎山、凤山万亩绿树环抱城区，万株大树荫蔽城区的绿化工程；建设北川河蓄水改造工程和边山边沟排洪体系，以河道蓄水美化城区的增加灵秀之气工程；扩大集中供热供气面积，治理严重污染源，实施净化空气质量的蓝天工程等三大类15项重点市政工程。全面改善城市环境质量，抓紧整治城市出入口两侧环境，给来吕之人形成良好的第一印象。同时，加快孝义、汾阳两市建设步伐，完善功能，培育产业，加强管理，提升品位，进一步提高城市的辐射带动能力。强化工作措施，加大支持力度，力争孝义市2007年之前跨入全国“百强县”行列。

稳步推进特色小城镇建设。适度超前、科学规划县城建设，综合推进排水、供水、供电、供热、供气、交通、通讯、污水处理、垃圾处理、防洪、绿化等基础配套工程建设，着力提升城镇的承载功能，下决心治理“脏、乱、差”，让每一个县城绿起来、净起来、亮起来、美起来。其他小城镇要按其文化底蕴、区位优势和产业特色，科学定位、完善设施、拓展功能，全面提高发展和建设水平。

（五）加强基础设施建设，突破加快发展的瓶颈制约

继续加快交通建设步伐。积极支持离军高速公路、307国道、209国道、340省道、沿黄干线公路、石清公路等国省道干线的建设和改造，完成沿黄公路一期184公里路基工程建设任务，改造离石至碛口旅游专线。探索农村公路养护新机制，加大养护投入，提高养护质量，着力解决群众行路难的问题。全力配合太中(银)铁路建设，全力推动岢瓦铁路、阳候铁路、太古岚铁路的建设步伐和孝柳铁路的改造和提升。抓好140个行政村3000公里的“村村通”水泥路建设工程，实现全部乡镇通油路、65%的行政村通水泥路（油路）、87%的行政村通客车的目标。

继续加大水利建设力度。千方百计落实横泉水库建设资金，做细做实移民搬迁工作，确保今年6月实现拦洪蓄水目标，使五十年的梦想变成现实。开工建设引文入川工程，积极筹划柏叶口水库工程。继续推进以水保生态体系建设和农村饮水安全工程为重点的水利基础设施建设。

继续加强电网建设。加快文水220千伏输变电工程建设进度，启动兴县、离石两个220千伏输变电工程和汾阳至中阳金罗、中阳金罗至柳林电厂两条220千伏双回路输电线路建设工程，积极争取500千伏输变电项目和岚县220千伏输变电工程开工建设。

（六）深层次推进改革开放，增强加快发展的活力

推动民营企业全面创新。支持“三大方阵”企业进行公司制改革，建立现代企业法人治理结构。引导一批有规模、有实力的民营企业引入职业经理队伍，建立有效的分配激励机制和约束机制，提高企业经营管理水平。激活民营企业人才市场，要有专门机构，为民营企业联络输送职业经营人才、专业技术人才，帮助解决人才匮乏的难题。支持重点民营企业进入资本市场，力争有2户民营企业在香港上市。继续加强对国有资产监督管理，逐户制定市属国有企业改制方案，筹措改制经费，排出时间进度，务求年内取得大的进展。

深化财政和金融体制改革。继续推进国库集中支付、部门预算、政府采购和收支两条线等财政综合改革，加强非税收入征收管理，建立“征收、预算、使用”三分离的管理体制。按照省政府调整省、市、县三级财政收支体制的要求，理顺财税收入分配关系，壮大县级财力，增强县级财政自我积累发展的能力。积极支持辖区内银行体制改革，创新金融产品，坚持政银企联席会议制度，加强项目推荐管理，搭建与各类商业银行特别是开行、农发行等政策性银行的合作平台，为重点项目建设进一步拓宽融资渠道。建立和完善市、县两级中小企业信用担保体系，引入贷款利率的风险定价机制，为中小企业提供便捷高效的信贷支持和金融服务。扎实推进农村信用社改革，充分发挥其服务“三农”的金融主力军作用。鼓励民间资本依法入股社区金融机构，积极培育小额贷款组织，解决农民群众贷款难的问题。

加大招商引资力度。坚持“开放促招商，招商促开发，开发促发展”的思路，以招商为主，引资为辅，重点抓好招商队伍建设和拓展招商市场两方面的工作。发挥政府、企业两大招商主体的作用，充分利用各类洽谈会平台，同国内外大型企业集团广泛接触，寻找合作的机会。认真研究发达地区的产业合作需求、产业转移趋向，积极寻求和引进一批上下游配套的综合开发项目，引进一批高技术含量、高附加值的骨干大项目，通过这些项目的示范扩散，吸引更多的客商和项目。各市县区及有关部门要精心筛选、包装项目，争取在省政府组织的赴香港、上海大型招商活动中取得较多的实质性成果。

（七）加快发展各项社会事业，统筹经济社会发展

加快发展教育事业。实施“双百”学校创建工程，建设452所农村寄宿制学校，巩固和提高“普九”成果。整合职业教育资源，扩大职业教育规模，筹建吕梁职业技术学院。推进中小学人事制度改革，加强教师、校长队伍建设，落实教师定期进修培训制度，重视教学研究，提高教育质量。加快吕梁市区教育发展步伐，集中力量抓好五项工作：一是合并贺昌中学东、西校区；二是扩大市实验中学办学规模，实行初、高中分离办学；三是支持大土河集团建设高标准小学、初中、高中各一所；四是开工建设吕梁学院(吕梁煤炭职业技术学院)；五是今明两年市财政各投入1000万元，支持离石区新建、改扩建一批中小学校。

大力推动科技创新。加快科技创新平台建设，围绕以煤化工为重点的支柱产业，依托国内外科研院所，发挥政府的引导作用和企业的创新主体作用，倾斜扶持科技研发中心和技术创新基地建设，增强我市的自主创新能力。大力引进和培育高新技术项目，培植科技型企业，推动全市科技进步。

加快发展卫生事业。加大农村卫生事业投入力度，整合卫生资源，加强业务培训，改善职工待遇，争取通过两年的努力使农村医疗卫生水平有一个较大提高。推进临县、孝义、柳林新型农村合作医疗制度试点工作。支持市人民医院住院大楼、汾阳医院外科楼的配套完善工作，真正把这两所医院建成设备先进、技术一流的区域性综合医院。

加强人才队伍建设。牢固确立人才资源是第一资源的观念，全面实施“新吕梁人才工程”。切实加强公务员管理，稳步推进事业单位人事制度改革。下大力气抓好招才引智工作，围绕培养、引进、留住、使用四个环节，努力建设好政务人才、企业经营管理人才、专业技术人才、高技能人才和农村实用人才队伍。特别要把人才引进和项目引进结合起来，通过项目吸纳、技术引进和人才租赁等方式，把更多的英才集聚到吕梁这块热土上来。

(八)着力解决涉及群众切身利益的问题，推进和谐社会建设

千方百计扩大就业。积极落实财政投入、小额贷款、免费培训和税费减免等促进再就业的优惠政策，强化就业服务体系建设，为城乡居民就业提供优质服务。国有企业下岗职工再就业政策期限延长三年，并根据各地实际把政策适用范围逐步扩大到城镇集体企业下岗职工。大力支持劳动密集型项目和服务行业，开发社区就业岗位和公益性就业岗位，千方百计增加就业岗位。同时把就业再就业与劳务输出紧密结合起来，努力扩大就业规模，力争使“零就业”家庭中至少有一人就业。

推进社会保障体系建设。深化养老保险制度改革，进一步拓宽资金筹措渠道，加强基金征管，提高养老保险金支付能力。统一城镇个体工商户和灵活就业人员的参保办法，着力提高不同所有制企业的参保率。完善城镇职工基本医疗和生育保险制度，扩大社会保险覆盖范围。完善城镇居民最低生活保障和农村社会保障制度，让每一位城乡困难群众基本生活得到保障。稳步推进农村养老、医疗保险等社会保障体系建设，构筑建设社会主义新农村的社保服务平台。

关注困难群众生活。进一步加大对城乡困难居民的帮扶力度，广泛开展生活、就业、就医、就学、住房、法律等十二项援助。强化劳动监察，严格执行用工最低工资制度。认真清理建设领域拖欠工程款和农民工工资问题，多还旧帐，不欠新帐。加快发展社会福利事业和残疾人事业，完善优抚保障机制和社会救助体系。

全力办好“十件实事”。群众利益无小事，一枝一叶总关情。市政府今年要集中财力、集中精力，为全市人民办好“十件实事”：一是继续实施扶贫攻坚工程，移民搬迁12200人，整村推进32710人。二是新增就业岗位223万个，培训农民15万，转移农村富余劳动力6万。三是继续实施农村中小学危房改造工程，落实农村家庭贫困学生“两免一补”政策，新建、改建城镇中小学标准操场105个。四是扩大农村新型合作医疗试点，试点县参合率达到80%，覆盖农业人口150万。五是加大乡镇卫生院医疗设备投入，全部配齐B超、X光、心电图“新三件”。六是继续实施城市集中供热工程，中心城市及县城新增131万平方米，逐步取缔供热锅炉和燃煤茶炉。七是继续实施“村村通”工程，新增140个行政村通水泥路（油路）、155个行政村通客车。八是抓好农村安全饮水工程建设，新解决20万农村人口的饮水安全问题。九是启动“万村千乡”市场工程，发展农村连锁便民店100家。十是全面兑现农村计划生育家庭四项奖励政策，推进“健苗工程”，降低人口出生率和出生缺陷率。

（九）加强民主法制和精神文明建设，维护社会稳定

推进依法治市。自觉接受人大及其常委会的法律监督、工作监督和政协的民主监督，广泛听取各民主党派、工商联和无党派人士的意见和建议，充分发挥工会、共青团、妇联等人民团体的桥梁纽带作用，支持法院、检察院依法履行职责。坚持依法行政，建立健全重大事项集体决策制度、专家咨询和评估制度、决策听证和公示制度，提高政府工作的透明度和公信力。积极推进“五五”普法教育，提高全民法律素质。依法保障公民特别是妇女、未成年人和残疾人的合法权益。继续加强基层民主法制建设，完善基层群众性自治组织和企事业单位民主管理制度，坚持政务、村务、厂务公开和民主选举制度，保证人民群众依法行使选举权、知情权、参与权和监督权。

加强精神文明建设。深入开展“八荣八耻”社会主义荣辱观教育，不断提高公民素质。积极推进文明城市、文明村镇等群众性精神文明创建活动。充分发挥纪念馆、博物馆的宣传教育作用，取缔黑网吧，加强未成年人思想道德建设，引导青少年健康成长。坚持多元化投资发展文化事业和文化产业，繁荣文学艺术、新闻出版、广播影视等文化事业，强化社会文化市场管理，营造弘扬科学、反对迷信的社会环境。抓好全民健身活动，提高群众健康水平。

切实维护社会稳定。完善突发性公共事件应急预案体系，建立健全应对各类突发性公共事件的工作机制，提高公共安全应急处置能力。大力推进社会治安防控体系建设，加强社会治安综合治理，高度重视信访工作和民族宗教，妥善处理人民内部矛盾。始终保持对敌斗争的高压态势，严厉打击各类刑事犯罪。进一步整顿和规范市场经济秩序，严厉打击制售假冒伪劣产品、商业欺诈等违法犯罪活动。

高度重视安全生产。构建“政府统一领导、部门依法监管、企业全面负责、群众监督参与、社会广泛支持”的安全生产工作格局。加强煤矿、非煤矿山、道路交通、人员聚集场所、民爆物品、危险化学品、食品药品、森林防火、动物防疫等领域的安全专项整治工作。要突出煤矿安全生产这一重点，进一步加大安全投入，严格落实安全生产工作责任制，切实加强安全监管，严厉打击非法、违法、违规生产和经营行为，扭转重特大安全事故多发的被动局面，确保人民群众生命财产安全。

（十）加强政府自身建设，树立“为民、务实、清廉”形象

保持勇于探索、奋发有为的精神状态。牢固树立强烈的进取意识和创新意识，以更加宽广的视野，学习借鉴发达地区的发展模式和经验。敏锐把握市场变化和国家政策导向，妥善处理发展过程中的矛盾，潜心研究事关改革发展稳定大局的问题，以更加开阔的思路，大胆探索切合吕梁实际的发展道路、工作方法、操作形式和运行方式。

建设勤政敬业、真抓实干的务实作风。大力弘扬求真务实、创业进取之风，坚决反对形式主义、官僚主义的不良作风，疲沓拖拉、得过且过、混日子的不良政风。强化责任意识，直面具体矛盾，解决实际问题，确保政策措施落实到位，工作任务一抓到底。强化政务纪律，加强部门之间的协调沟通，确保政令畅通。强化目标管理，确保目标分解到位、压力传递到位、工作落实到位。强化行政效能监察和责任追究，各级政府要善于抓战略重点和关键环节，周密安排，精心实施，确保取得实效。严厉查处失职、渎职行为。

树立公开简捷、高效便民的服务理念。始终把最广大人民群众的根本利益作为政府工作的出发点和落脚点，强化公仆意识和宗旨观念，把保持共产党员先进性教育的实际成效体现在关心群众冷暖，全心全意为群众办实事、做好事、解难题上来。进一步转变政府职能，深化行政审批制度改革，把该减的减到位、该放的放彻底，为群众提供“一条龙”、“一站式”优质服务，着力解决政府权力部门化、部门权力个人化以及群众办事难、行政成本高的问题。加快发展电子政务，建立完善政府门户网站，搭建政务公开、资源共享的信息平台，畅通社会监督渠道，增强政府工作透明度，促进政府服务水平进一步提高。

打造依法行政、廉洁从政的清廉形象。全面落实行政执法责任制和领导干部任期经济责任审计制，切实加强行政监督，认真治理商业贿赂，严厉查处违法违纪案件。坚决纠正行业不正之风，重点查处征地拆迁、企业改制、医疗服务、教育收费、企业“三乱”、公路“三乱”等方面侵害群众利益的行为，切实维护群众的合法权益。政府公务人员特别是领导干部要模范遵守“四大纪律”，自觉落实“八项要求”，时刻牢记“五个不许”，甘于吃苦，乐于奉献，耐得住清贫，挡得住诱惑，始终保持堂堂正正做人、清清白白为官、干干净净办事的高尚情操，真正把“亲民如子、敬民如父”的从政理念融入到工作实践中，努力在人民群众中树立执政为民、廉洁从政的良好形象。

各位代表，时代赋予我们重任，人民寄予我们厚望。喜看今朝，全市人民正在谱写全面崛起的壮丽篇章；展望明天，吕梁大地将呈现富裕、文明、和谐的美好前景。让我们更加紧密地团结在以胡锦涛同志为总书记的党中央周围，在中共吕梁市委的坚强领导下，锐意进取，开拓创新，励精图治，扎实工作，为建设经济繁荣、人民富裕、社会和谐、环境友好的新吕梁而努力奋斗！
